# Supplementary material for: Dietary Phytase and Lactic Acid-Treated Cereal Grains Differently Affected Calcium and Phosphorus Homeostasis from Intestinal Uptake to Systemic Metabolism in a Pig Model
Source: Nutrients. 2020 May 25;12(5):1542. doi: 10.3390/nu12051542 (PMC7284645; doi:10.3390/nu12051542)
Supplement: Supplementary file 1 [file nutrients-12-01542-s001.pdf]

nutrients

Supplementary

Table S1. Oligonucleotide primers used for quantitative PCR.

Genes[1]	Accession number[2]	Primer sequence (5′ to 3′)	Amplification efficiencies	Amplicon size (bp)	Reference[3]
SLC34A1	NM_001044623.1	F: TCAACTCTCTGCTCAAGGGC<br>R: CACCTAGGCCAATGAGTGGG	94.0	183	newly designed
SLC34A2	NM_001256772.1	F: CGTGTTCTCGTCGACTCTGA<br>R: CCAGCGGTACTTGGATGAGAT	90.0	280	newly designed
TRPV5	XM_021078896.1, XR_002340352.1	F: TCCCTGTAACTTGCCAGTGC<br>R: TGCTGATCCCAGTCTTGCTG	99.7	103	newly designed
TRPV6	FJ268731.2	F: GAATGCGGTTGCATTGAGCA<br>R: TTACACCCTTTCCACAGCCG	89.8	112	newly designed
CALB1	NM_001130226.1	F: ATTTCGACGCTGACGGAAGT<br>R: TTGCTGGCATCGGAATAGCA	94.1	224	newly designed
PMCA1b	X53456.1, XR_002343820.1, XM_021091182.1, NM_214352.3	F: GAAAATGGTTCCTGCTGCC<br>R: GCAACCGAGTTGTTTGCCAT	96.4	275	newly designed
VDR	NM_001097414.1	F: TGGTTGGAAGTGTCTGGGAG<br>R: GGGGTCAGGTAAGGAAGTGC	99.8	117	newly designed
CYP24A1	NM_214075.2	F: TTGGGTTCGTTCGACTCCG<br>R: TCCACGGTTTGATCTCCAGC	90.6	103	newly designed
CYP27B1	NM_213995.1, XM_021091124.1	F: TCATGGGTGGCTGAGGAAGAA<br>R: ATAACTGTTTAGCCAGCAGCAC	97.2	86	newly designed
FGF23	XM_001926525.4	F: CGCAGGCTTCGTGGTCATAA<br>R: GGTACACGTCGTAGCCGTTC	98.9	146	newly designed
OCN	AY150038.1, XM_013996978.2, NM_001164004.1	F: ACGAGCTGGCTGATCACATC<br>R: CTGCGAGGTCTAGGCTATGC	94.2	71	newly designed
OPG	XM_003481346.4	F: GACCTCTGTGAAAACAGTGTGC<br>R: CTTCAGACTCTGTGTGACGGT	111.3	276	newly designed
RANKL	AY606802.1, XM_001925694.6	F: CCAAGACGCCACGTACTTT<br>R: AAACAACTCCACAAAATGGGGC	93.4	71	newly designed
CDH1	NM_001163060.1	F: TACCTGAACGAGTGGGGCAA<br>R: CCCATCACATGAGCGTAGGG	107.0	118	[1]
CLDN1	NM_001244539.1	F: TGATGAGGTGCAGAAGATGC<br>R: CCATGCTGTGGCAACTAAGA	91.4	88	[1]
CLDN4	NM 001161637.1	F: CAACTGCGTGGATGATGAGA<br>R: CCAGGGGATTGTAGAAGTCG	90.1	140	[1]
OCLN	NM_001163647.1	F: TTGTGGGACAAGGAACGTATTTA<br>R: TGCCTGCCGACACGTTT	92.0	76	[1]
ZO1	XM_003353439.2	F: AAGCCCTAAGTTCAATCACAATCT<br>R: ATCAAACTCAGGAGGCGGC	103.1	130	[1]
MUC2	XM_013989745.1	F: TGCCATCTACACCAAGGTCTATTC<br>R: TCTGCAGGCCGTTGTAGTCTC	90.6	137	[1]
MUC4	XM_001926442.1	F: GATGCCCTGGCCACAGAA<br>R: TGATTCAAGGTAGCATTCATTTGC	97.9	89	[1]
ACTG	XM_003357928.4	F: GGGCATCCTGACCCTCAAG<br>R: TGTAGAAGGTGTGATGCCAGATCT	96.7	89	[1]
GAPDH	NM_001206359.1	F: GGCGTGAACCATGAGAAGTATG<br>R: GGTGCAGGAGGCATTGCT	97.0	60	[2]
B2M	NM_213978.1	F: CCCCCGAAGGTTCAGGTT<br>R: GCAGTTCAGGTAATTTGGCTTTC	100.6	66	[2]
HPRT	NM_001032376.2	F: AGAAAAGTAAGCAGTCAGTTTCATATCAGT<br>R: ATCTGAACAAGAGAGAAAATACAGTCAATAG	92.3	131	[2]
OAZ1	NM_001122994.1	F: TCGGCTGAATGTAACAGAGGAA<br>R: GAGCCTGGATTGGACGTTTAAA	96.1	70	[2]

[1] *SLC34A1*, Na+-Pi cotransporter 1; *SLC34A2*, Na+-Pi cotransporter 2; *TRPV5*, transient receptor potential vanilloid 5; *TRPV6*, transient receptor potential vanil-loid 6; *CALB1*, calbindin; *PMCA1b*, plasma membrane Ca2+ adenosintriphosphatase; *VDR*, vitamin D receptor; ; *CYP24A1*, cytochrome P450, family 24, subfam-ily A,

polypeptide 1; *CYP27B1*, cytochrome P450, family 24, subfamily B, polypeptide 1; *FGF23*, fibroblast growth factor 23; *OCN*, osteocalcin; *OPG*, osteo-protegrin; *RANKL*, receptor activator of NF-κB ligand; *CDH1*, cadherin-1; *CLDN1*, claudin-1; *CLDN4*, claudin-4; *OCLN*, occludin; *ZO1*, zona occludens-1; *MUC2*, mucin 2; *MUC4*, mucin 4; *ACTB*, β-actin; *GAPDH*, glyceraldehyde-3-phosphate-dehydrogenase; *B2M*, β2-microglobulin; *HPRT*, hypoxanthin-guanine phosphoribosyl transferase; *OAZ1*, ornithine decarboxylase antizyme.

[2] National Center for Biotechnology Information (NCBI) (http://www.ncbi.nlm.nih.gov/sites/entrez?db=gene).

[3] [1] Klinsoda, J.; Vötterl, J.; Zebeli, Q.; Metzler-zebeli, B.U. Alterations of the viable ileal microbiota of gut-mucosa-lymph node axis in pigs fed phytase and lactic acid-treated cereals. Appl. Environ. Microbiol. 2020, 86, e02128.

[2] Metzler-Zebeli, B.U.; Ertl, R.; Grüll, D.; Molnar, T.; Zebeli, Q. Enzymatically modified starch up-regulates expression of incretins and sodium-coupled monocarboxylate transporter in jejunum of growing pigs. Animal 2017, 11, 1180–1188.

Table S2. Effect of dietary phytase and lactic acid-treated cereals on performance parameters.

Parameter[2]	Dietary treatment[1]				SEM	*p* - Value		
	Con	LA	Con-Phytase	LA-Phytase		Phytase	LA	Phytase × LA
BW start (kg)	13.3	13.1	13.1	13.1	0.64	0.832	0.862	0.832
BW final (kg)	23.6	24.3	23.7	24.3	0.95	0.948	0.510	0.938
ADG (g/d)	555	609	578	607	35.1	0.772	0.251	0.726
ADFI (g/d)	779	771	814	790	20.2	0.192	0.430	0.675
FCR	1.40	1.33	1.41	1.30	0.051	0.856	0.089	0.727

[1] Experimental period of 19 days; Con, control; LA, lactic acid treated cereals, Phytase 500 FTU/ kg diet (VM Phytase XP 897420).

[2]Values are presented as least square means ± SEM; n = 8/diet; Performance data were calculated for the time period experimental day 0 to experimental day 18/ 19; BW, body weight; BWG, body weight gain; ADG, average daily gain; ADFI, average daily feed intake; FCR, feed conversion ratio (kg feed/kg body weight gain)

Table S3. Effect of dietary phytase and lactic acid-treated cereals on phosphorus, calcium and nitrogen balance and ratio of calcium to phosphorus balance.

Parameter[2]	Dietary treatment[1]				SEM	*p* - Value		
	Con	LA	Con-Phytase	LA-Phytase		Phytase	LA	Phytase × LA
			P balance (g/d)					
P intake	4.54	4.34	4.66	4.39	0.058	0.168	<0.001	0.498
Total P excretion	1.94	1.64	1.37	1.08	0.079	<0.001	0.001	0.950
Faecal P excretion	1.89	1.60	1.16	0.92	0.066	<0.001	<0.001	0.731
Urinary P excretion	0.06	0.05	0.20	0.16	0.028	<0.001	0.366	0.642
P absorption	2.65	2.75	3.50	3.47	0.079	<0.001	0.697	0.429
P retention	2.60	2.70	3.30	3.31	0.087	<0.001	0.515	0.580
			P balance (% of daily intake)					
P total excretion	42.76	37.91	29.21	24.38	1.631	<0.001	0.007	0.994
P faecal excretion	41.53	36.85	24.91	20.70	1.398	<0.001	0.004	0.868
P urine excretion	1.23	1.06	4.30	3.68	0.575	<0.001	0.501	0.702
P absorption	58.47	63.15	75.09	79.30	1.398	<0.001	0.004	0.868
P retention	57.24	62.09	70.79	75.62	1.631	<0.001	0.007	0.994
			Ca balance (g/d)					
Ca intake	5.49	5.45	5.57	5.46	0.069	0.460	0.292	0.623
Total Ca excretion	3.37	2.85	2.15	1.84	0.135	<0.001	0.005	0.443
Faecal Ca excretion	1.48	1.26	0.99	0.93	0.071	<0.001	0.050	0.263
Urinary Ca excretion	0.85	1.42	0.12	0.16	0.077	<0.001	0.001	0.002
Ca absorption	4.00	4.19	4.58	4.54	0.096	<0.001	0.469	0.250
Ca retention	3.15	2.77	4.46	4.38	0.142	<0.001	0.119	0.300
			Ca balance (% of daily intake)					
Ca total excretion	61.28	52.55	38.53	33.34	2.327	<0.001	0.006	0.455
Ca faecal excretion	26.87	23.06	17.67	16.70	1.231	<0.001	0.063	0.258
Ca urine excretion	15.64	26.27	2.20	2.91	1.379	<0.001	<0.001	0.001
Ca absorption	73.13	76.94	82.33	83.30	1.231	<0.001	0.063	0.258
Ca retention	57.49	50.68	80.14	80.39	2.258	<0.001	0.159	0.130
			N balance (g/d)					
N intake	28.19	28.38	29.08	29.05	0.405	0.064	0.848	0.787
Total N excretion	8.30	8.22	8.57	7.66	0.356	0.681	0.180	0.251
Faecal N excretion	3.21	3.16	3.30	3.03	0.124	0.881	0.211	0.399
Urinary N excretion	5.09	5.07	5.27	4.63	0.383	0.741	0.394	0.421
N absorption	24.98	25.22	25.79	26.02	0.394	0.053	0.554	0.990
N retention	19.89	20.15	20.52	21.39	0.436	0.043	0.205	0.490
			N balance (% of daily intake)					
N total excretion	29.42	28.69	29.38	26.26	1.152	0.294	0.108	0.310
N faecal excretion	11.44	11.06	11.29	10.43	0.432	0.376	0.164	0.581
N urine excretion	17.98	17.63	18.09	15.83	1.268	0.511	0.315	0.459
N absorption	88.56	88.94	88.71	89.57	0.432	0.376	0.164	0.581
N retention	70.58	71.31	70.62	73.74	1.152	0.294	0.108	0.310
			Ratio of calcium to phosphorus balance					
Ratio of ingested Ca/P	1.21	1.26	1.20	1.25	0.005	0.062	<0.001	0.630
Ratio of totally excreted Ca/P[3]	1.43	1.39	1.32	1.37	0.029	0.032	0.915	0.109
Ratio of excreted Ca/P in faeces[3]	0.65	0.63	0.70	0.81	0.022	<0.001	0.057	0.010
Ratio of excreted Ca/P in urine[3]	16.98	26.87	0.93	0.83	2.198	<0.001	0.035	0.032
Ratio of absorbed Ca/P[3]	1.25	1.23	1.10	1.05	0.013	<0.001	0.010	0.510
Ratio of retained Ca/P[3]	1.01	0.81	1.13	1.06	0.025	<0.001	<0.001	0.018

[1] Experimental period of 19 days; Con, control; LA, lactic acid treated cereals.

[2]Values are presented as least square means ± SEM; n = 8/diet. The nutrient intake, nutrient excretion in faeces and urine as well as absorption and retention were calculated as the mean of the three days of sampling (experimental day 15 to day 17).

[3] Ratio calculated with data stated as a percentage per daily intake.

Table S4. Effect of dietary phytase and lactic acid-treated cereals on relative expression of genes (fold change) related to phosphorus and calcium absorption along the intestinal tract.

Parameter[2]	Gut Site	Dietary treatment[1]				SEM	p - Value		
		Con	LA	Con-Phytase	LA-Phytase		Phytase	LA	Phytase × LA
CDH1	Duodenum	0.05	0.05	0.05	0.05	0.004	0.774	0.960	0.168
	Jejunum	0.40	0.43	0.40	0.52	0.052	0.371	0.159	0.435
	Ileum[3]	0.03	0.03	0.01	0.02	0.005	0.012	0.336	0.505
	Caecum	0.09	0.10	0.08	0.08	0.005	0.037	0.089	0.685
	Colon	0.11	0.11	0.11	0.09	0.006	0.276	0.082	0.068
CLDN1	Duodenum	0.04	0.02	0.04	0.04	0.008	0.496	0.511	0.198
	Jejunum	0.25	0.24	0.39	0.31	0.071	0.171	0.541	0.640
	Ileum[3]	0.08	0.09	0.10	0.05	0.017	0.535	0.231	0.152
	Caecum	0.03	0.02	0.02	0.02	0.005	0.682	0.334	0.440
	Colon	0.04	0.05	0.06	0.06	0.013	0.222	0.692	0.617
CLDN4	Duodenum	0.04	0.03	0.05	0.05	0.006	0.080	0.941	0.586
	Jejunum	0.43	0.33	0.50	0.39	0.052	0.229	0.053	0.961
	Ileum[3]	0.02	0.02	0.02	0.01	0.004	0.841	0.408	0.163
	Caecum	0.02	0.02	0.02	0.02	0.003	0.157	0.578	0.867
	Colon	0.03	0.02	0.03	0.02	0.004	0.850	0.088	0.736
OCLN	Duodenum	0.02	0.02	0.02	0.02	0.002	0.392	1.000	0.073
	Jejunum	0.43	0.46	0.46	0.57	0.056	0.198	0.213	0.449
	Ileum[3]	0.02	0.02	0.02	0.01	0.003	0.109	0.742	0.202
	Caecum	0.02	0.02	0.02	0.02	0.002	0.879	0.260	0.939
	Colon	0.04	0.04	0.03	0.03	0.002	0.041	0.257	0.257
ZO1	Duodenum	0.05	0.03	0.04	0.04	0.006	0.686	0.364	0.302
	Jejunum	0.41	0.36	0.29	0.52	0.070	0.754	0.207	0.060
	Ileum[3]	0.03	0.03	0.04	0.03	0.011	0.940	0.805	0.922
	Caecum	0.07	0.09	0.07	0.08	0.004	0.172	0.001	0.473
	Colon	0.09	0.10	0.09	0.07	0.005	0.043	0.372	0.037
MUC2	Duodenum	0.09	0.10	0.07	0.08	0.013	0.075	0.731	0.880
	Jejunum	0.37	0.42	0.42	0.54	0.066	0.221	0.203	0.526
	Ileum[3]	0.05	0.09	0.06	0.04	0.012	0.195	0.615	0.049
	Caecum	0.08	0.09	0.07	0.07	0.006	0.019	0.582	0.617
	Colon	0.17	0.19	0.14	0.13	0.017	0.015	0.850	0.385
MUC4	Duodenum	0.003	0.005	0.001	0.006	0.0027	0.733	0.243	0.689
	Jejunum	0.018	0.020	0.006	0.019	0.0059	0.267	0.213	0.336
	Ileum[3]	0.01	0.03	0.01	0.01	0.007	0.423	0.287	0.387
	Caecum	0.51	0.63	0.36	0.38	0.064	0.005	0.262	0.416
	Colon	0.44	0.63	0.66	0.46	0.062	0.673	0.936	0.005

[1] Experimental period of 19 days; Con, control; LA, lactic acid treated cereals, Phytase 500 FTU/ kg diet (VM Phytase XP 897420).

[2]Values are presented as least square means ± SEM; *n* = 8/diet; *CDH1*, cadherin-1; *CLDN1*, claudin-1; *CLDN4*, claudin-4; *OCLN*, occludin; *ZO*1, zona occludens-1; *MUC2*, mucin 2; *MUC4*, mucin 4.

[3] Relative gene expression data for innate immune genes from the ileum were already published in Klinsoda et al. 2020 (Klinsoda, J.; Vötterl, J.; Zebeli, Q.; Metzler-zebeli, B.U. Alterations of the viable ileal microbiota of gut-mucosa-lymph node axis in pigs fed phytase and lactic acid-treated cereals. Appl. Environ. Microbiol. 2020, 86, e02128.)

Table S5. Pearson's correlations coefficients between serum, bone parameters, and relative gene expression in the metacarpal bone and in the kidney (* $p < 0.05$; $|r| > 0.035$).

		Serum parameters								
		Calcium (mmol/L)	Phosphorus (mmol/L)	Serum Ca/P	Serum Ca×P	Vitamin D (ng/ml)	FGF23 (pg/ml)	Osteocalcin (µg/ml)	Parathormon (pg/ml)	Alkaline phosphatase (U/L)
**Diet**	Ca/P ratio	0.10	-0.15	0.26	0.03	0.02	0.37*	0.11	-0.23	-0.03
**Serum parameters**	Calcium (mmol/L)	1.00	-			-	-	-	-	-
	Phosphorus (mmol/L)	0.53*	1.00			-	-	-	-	-
	Serum Ca/P	0.50*	-0.45*	1.00						
	Serum Ca×P	0.88*	0.86*	0.04	1.00					
	Vitamin D (ng/ml)	-0.08	0.12	-0.20	0.02	1.00	-	-	-	-
	FGF23 (pg/ml)	0.47*	-0.02	0.48*	0.29	-0.02	1.00	-	-	-
	Osteocalcin (µg/ml)	-0.02	0.07	-0.11	0.03	0.22	0.31	1.00	-	-
	Parathormon (pg/ml)	-0.01	0.17	-0.19	0.11	0.26	0.03	0.41*	1.00	-
	Alkaline phosphatse (U/L)	0.66*	0.48*	0.21	0.64*	0.17	0.37*	0.07	0.12	1.00
**Metric parameters in metacarpal bone**	right metacarpal length (mm)	-0.28	-0.22	-0.07	-0.28	-0.22	-0.28	-0.26	0.40*	-0.34
	right metacarpal weight (g)	-0.40*	-0.36*	-0.05	-0.41*	0.19	-0.09	0.27	0.29	-0.48*
	cortical wall thickness (mm)	0	0.03	-0.01	0.02	-0.09	-0.21	0.03	-0.13	0.05
	cross sectional area (mm2)	-0.04	0.01	-0.01	-0.02	-0.06	-0.22	-0.03	-0.12	-0.02
	cortical index	0.10	0.09	0.02	0.11	-0.12	-0.11	0.15	-0.12	0.19
	density (g/cm3)	-0.14	0.11	-0.28	-0.01	-0.05	-0.10	0.10	0.28	0.002
	DM (%)	-0.04	0.11	-0.14	0.02	0.03	-0.14	-0.16	0.20	-0.18
	CA (%)	-0.32	0.21	-0.55*	-0.06	0.46*	-0.17	-0.04	0.40*	-0.15
	Calcium oxide (% of ash)	0.01	0.05	-0.02	-0.04	-0.09	-0.20	-0.09	-0.17	-0.05
	Phosphorus pentoxide (% of ash)	0.13	0.17	0	0.09	0.01	-0.35*	-0.19	-0.40*	-0.04
**Relative gene expression in metacarpal bone**	*CYP24A1*	-0.03	-0.21	0.18	-0.14	-0.39*	-0.27	-0.17	0	-0.25
	CYP27B1	0.02	-0.01	0.01	-0.01	-0.39*	-0.12	-0.30	0.30	-0.23
	VDR	-0.12	-0.15	0.04	-0.16	-0.05	-0.12	-0.25	0.03	-0.03
	FGF23	-0.17	0.07	-0.25	-0.06	0.14	-0.17	-0.03	0.80*	-0.14
	OCN	0.04	-0.22	0.27	-0.11	-0.34	0.05	-0.28	-0.35*	-0.07
	OPG	-0.07	-0.14	0.06	-0.13	-0.33	-0.17	-0.33	-0.01	-0.21
	RANKL	-0.09	-0.03	-0.08	-0.09	-0.37*	-0.23	-0.35*	-0.19	-0.28
**Relative gene expression in the kidney**	*SLC34A1*	-0.06	-0.15	0.06	-0.12	0.30	0.34	0.09	0.13	0.13
	TRPV5	-0.34	0.34	-0.73*	0	0.36*	-0.28	0.02	0.21	-0.22
	TRPV6	-0.11	-0.24	0.10	-0.17	-0.11	0.46*	0.37*	0.13	-0.16
	CALB1	-0.31	0.34	-0.69*	0	0.48*	-0.28	0.03	0.02	-0.09
	PMCA1b	-0.12	-0.16	0.04	-0.16	0.05	0.40*	-0.03	-0.28	0.05
	VDR	-0.06	0.43*	-0.50*	0.20	0.57*	-0.26	0	0.27	0.03
	CYP24A1	0.31	0.12	0.19	0.28	0.13	0.35	0.50*	-0.13	0.13

Table S6. Pearson's correlations coefficients between diet, bone parameters, and relative expression of genes related to phosphorus and calcium metabolism in the metacarpal bone and kidney (* p < 0.05; |r|> 0.035).

		Diet	Metric parameters in metacarpal bones										Relative gene expression in metacarpal bone							Relative gene expression in the kidney						
		Ca/P ratio	right metacarpal length (mm)	right metacarpal weight (g)	cortical wall thickness (mm)	cross sectional area (mm2)	cortical index	density (g/cm3)	DM (%)	Ash (%)	Calcium oxide (% of ash)	Phosphorus pentoxide (% of ash)	*CYP24A1*	*CYP27B1*	*VDR*	*FGF23*	*OCN*	*OPG*	*RANKL*	*SLC34A1*	*TRPV5*	*TRPV6*	*CALB1*	*PMCA1b*	*VDR*	*CYP24A1*
Diet	Ca/P ratio	1.00	-	-	-	-	-	-	-	-	-	-	-	-	-	-	-	-	-	-	-	-	-	-	-	-
Metric parameters in metacarpal bones	right metacarpal length (mm)	0.16	1.00	-	-	-	-	-	-	-	-	-	-	-	-	-	-	-	-	-	-	-	-	-	-	-
	right metacarpal weight (g)	-0.15	0.01	1.00	-	-	-	-	-	-	-	-	-	-	-	-	-	-	-	-	-	-	-	-	-	-
	cortical wall thickness (mm)	-0.05	0.20	0.14	1.00	-	-	-	-	-	-	-	-	-	-	-	-	-	-	-	-	-	-	-	-	-
	cross sectional area (mm2)	-0.06	0.20	0.13	0.96*	1.00	-	-	-	-	-	-	-	-	-	-	-	-	-	-	-	-	-	-	-	-
	cortical index	-0.05	0.13	0.12	0.91*	0.78*	1.00	-	-	-	-	-	-	-	-	-	-	-	-	-	-	-	-	-	-	-
	density (g/cm3)	0.11	0.03	0.03	-0.13	-0.17	-0.04	1.00	-	-	-	-	-	-	-	-	-	-	-	-	-	-	-	-	-	-
	DM (%)	-0.32	0.21	-0.04	-0.12	-0.10	-0.09	-0.31	1.00	-	-	-	-	-	-	-	-	-	-	-	-	-	-	-	-	-
	Ash (%)	-0.22	0.00	-0.03	-0.12	-0.10	-0.15	-0.09	0.57*	1.00	-	-	-	-	-	-	-	-	-	-	-	-	-	-	-	-
	Calcium oxide (% of ash)	-0.50*	-0.35*	0.16	-0.04	-0.01	-0.07	-0.40*	0.25	0.07	1.00	-	-	-	-	-	-	-	-	-	-	-	-	-	-	-
	Phosphorus pentoxide (% of ash)	-0.46*	-0.28	0.03	-0.07	-0.04	-0.06	-0.17	0.30	0.05	0.70*	1.00	-	-	-	-	-	-	-	-	-	-	-	-	-	-
Relative gene expression expressed in	*CYP24A1*	-0.17	0.12	-0.05	0.01	-0.07	0.12	0.08	-0.06	-0.31	-0.03	0.09	1.00	-	-	-	-	-	-	-	-	-	-	-	-	-
	CYP27B1	-0.18	0.25	-0.08	-0.04	-0.11	0.08	-0.05	0.23	-0.04	0.17	0.05	0.66*	1.00	-	-	-	-	-	-	-	-	-	-	-	-
	VDR	-0.34	0.02	-0.11	0.07	0.05	0.06	-0.13	0.43*	0.34	0.25	0.11	-0.01	0.18	1.00	-	-	-	-	-	-	-	-	-	-	-
	FGF23	-0.34	0.28	0.16	-0.25	-0.28	-0.16	0.15	0.28	0.30	-0.09	0	0.39*	0.48*	0.13	1.00	-	-	-	-	-	-	-	-	-	-
	OCN	-0.16	0.08	0.01	0.24	0.15	0.33	-0.15	0.21	-0.02	0.23	-0.02	0.40*	0.67*	0.46*	0.12	1.00	-	-	-	-	-	-	-	-	-
	OPG	-0.23	0.13	-0.04	0.14	0.05	0.26	-0.06	0.21	0.05	0.23	0.06	0.62*	0.83*	0.41*	0.35*	0.89*	1.00	-	-	-	-	-	-	-	-
	RANKL	-0.24	0.21	-0.07	-0.02	-0.10	0.08	-0.05	0.20	0.01	0.12	0.05	0.71*	0.92*	0.29	0.49*	0.69*	0.87*	1.00	-	-	-	-	-	-	-
Relative gene expression in the kidney	*SLC34A1*	0.33	0.02	-0.03	-0.10	-0.10	-0.08	0.28	-0.05	0.23	-0.40*	-0.37*	-0.13	-0.15	-0.05	0.02	-0.10	-0.20	-0.18	1.00	-	-	-	-	-	-
	TRPV5	-0.08	0.09	-0.07	-0.28	-0.23	-0.31	0.21	0.31	0.62*	0	0.12	-0.30	-0.08	0.01	0.19	-0.39*	-0.21	-0.08	0.03	1.00	-	-	-	-	-
	TRPV6	0.28	0.01	0.29	0.04	0.04	0.04	0	-0.18	-0.05	-0.26	-0.47*	-0.02	-0.06	-0.17	0.04	0.12	-0.04	-0.14	0.25	-0.17	1.00	-	-	-	-
	CALB1	-0.08	-0.05	-0.06	-0.01	0.02	-0.04	0.28	0.21	0.65	0	0.14	-0.29	-0.20	-0.02	0.09	-0.36*	-0.22	-0.17	0.33	0.83*	-0.17	1.00	-	-	-
	PMCA1b	0.23	-0.13	-0.05	-0.12	-0.07	-0.18	0.18	-0.09	0.16	-0.18	-0.21	-0.25	-0.22	0	-0.26	0	-0.19	-0.18	0.77*	0.01	0.25	0.21	1.00	-	-
	VDR	-0.08	0.14	-0.13	-0.17	-0.12	-0.22	0.23	0.27	0.61*	-0.08	0.19	-0.32	-0.21	-0.05	0.22	-0.37*	-0.25	-0.20	0.19	0.67*	-0.31	0.70*	0.08	1.00	-
	CYP24A1	0.64*	-0.11	-0.03	0.09	0.07	0.12	0.20	-0.25	-0.18	-0.35*	-0.24	-0.28	-0.39*	-0.32	-0.41*	-0.23	-0.41*	-0.44*	0.14	-0.02	0.26	-0.001	0.07	0.07	1.00
